# Supplementary material for: Integrated analyses of multi-omic data derived from paired primary lung cancer and brain metastasis reveal the metabolic vulnerability as a novel therapeutic target
Source: Genome Med. 2024 Nov 26;16:138. doi: 10.1186/s13073-024-01410-8 (PMC11590298; doi:10.1186/s13073-024-01410-8)
Supplement: Supplementary file 2 — Additional file 2: Fig. S1: Analysis pipeline for integrated multi-omics data in this study. Fig. S2: Mutation landscape in paired primary lung cancer and brain metastases. Fig. S3: Comparative tumor mutational burden in lung cancer and brain metastases. Fig. S4: Copy number alterations in primary lung cancer versus brain metastases. Fig. S5: Principal component analysis and pathway enrichment in lung cancer brain metastasis. Fig. S6: Immune pathway correlations and lymphocyte composition in lung cancer brain metastases. Fig. S7: Enrichment analysis of mitochondrial and immune pathways in independent brain metastasis cohort. Fig. S8: Immunohistochemical profiling of proliferation and immune markers in brain metastases. [file 13073_2024_1410_MOESM2_ESM.pdf]

## Supplementary Information

**Additional file 2:** Supplementary Materials for: Integrated Analyses of Multi-omic Data Derived from Paired Primary Lung Cancer and Brain Metastasis Reveal the Metabolic Vulnerability as a Novel Therapeutic Target. This file includes Supplementary Figures: Fig. S1-S8


**Fig. S1:** Analysis pipeline for integrated multi-omics data in this study.
**Fig. S2:** Mutation landscape in paired primary lung cancer and brain metastases.
**Fig. S3:** Comparative tumor mutational burden in lung cancer and brain metastases.
**Fig. S4:** Copy number alterations in primary lung cancer versus brain metastases.
**Fig. S5:** Principal component analysis and pathway enrichment in lung cancer brain metastasis.
**Fig. S6:** Immune pathway correlations and lymphocyte composition in lung cancer brain metastases.
**Fig. S7:** Enrichment analysis of mitochondrial and immune pathways in independent brain metastasis cohort.
**Fig. S8:** Immunohistochemical profiling of proliferation and immune markers in brain metastases.

# Supplemental Figure 1

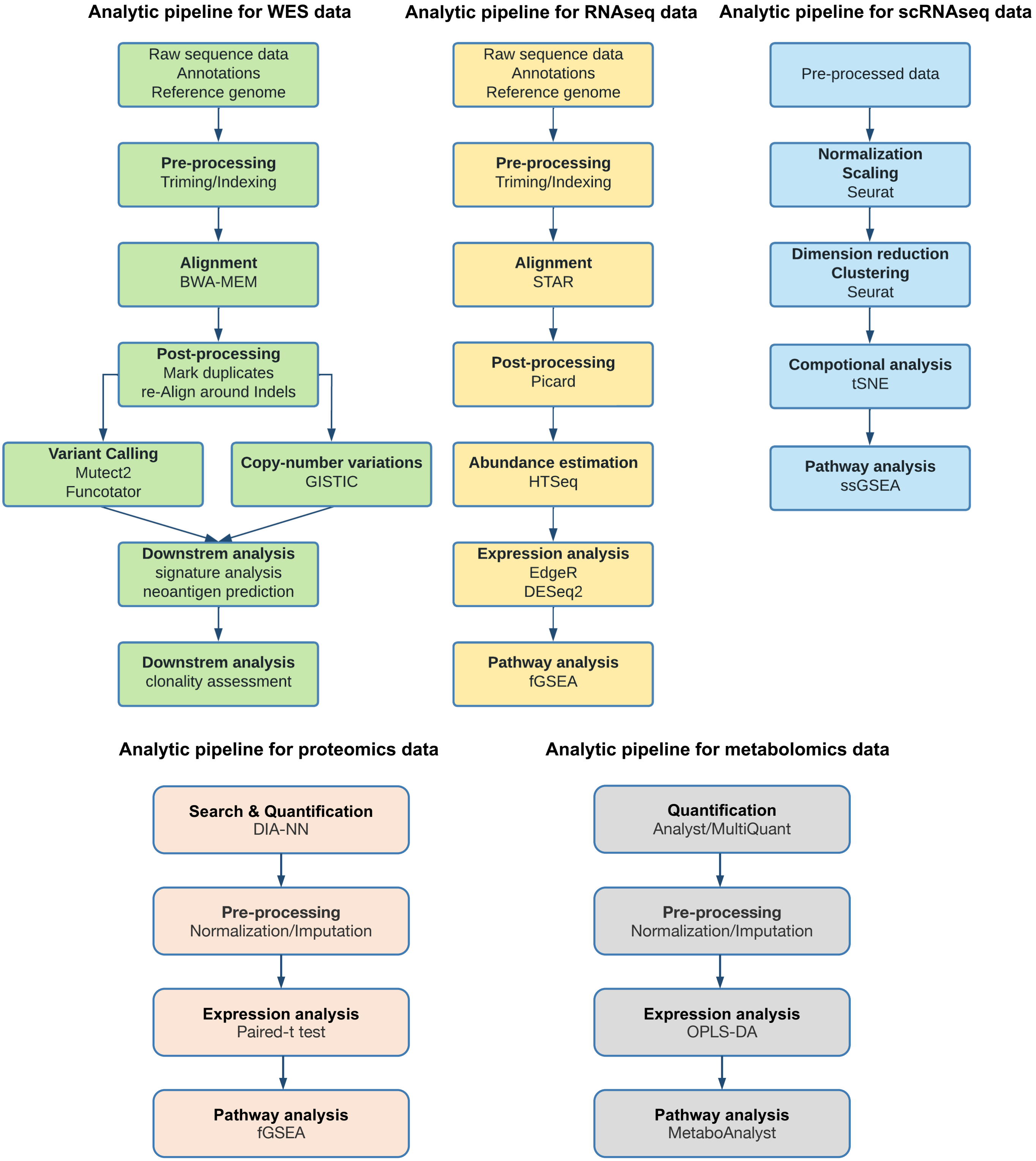


**Fig. S1. Related to Figure 1. Analysis pipeline for integrated multi-omics data in this study.** Analysis pipeline for whole-exome sequencing (WES), RNA sequencing (RNAseq), single cell RNA sequencing (scRNAseq), proteomics, and metabolomics data. Flow-chart demonstrates sequential use of tools in evaluation of DNA, RNA, single cell RNA, protein and metabolite samples.

# Supplemental Figure 2

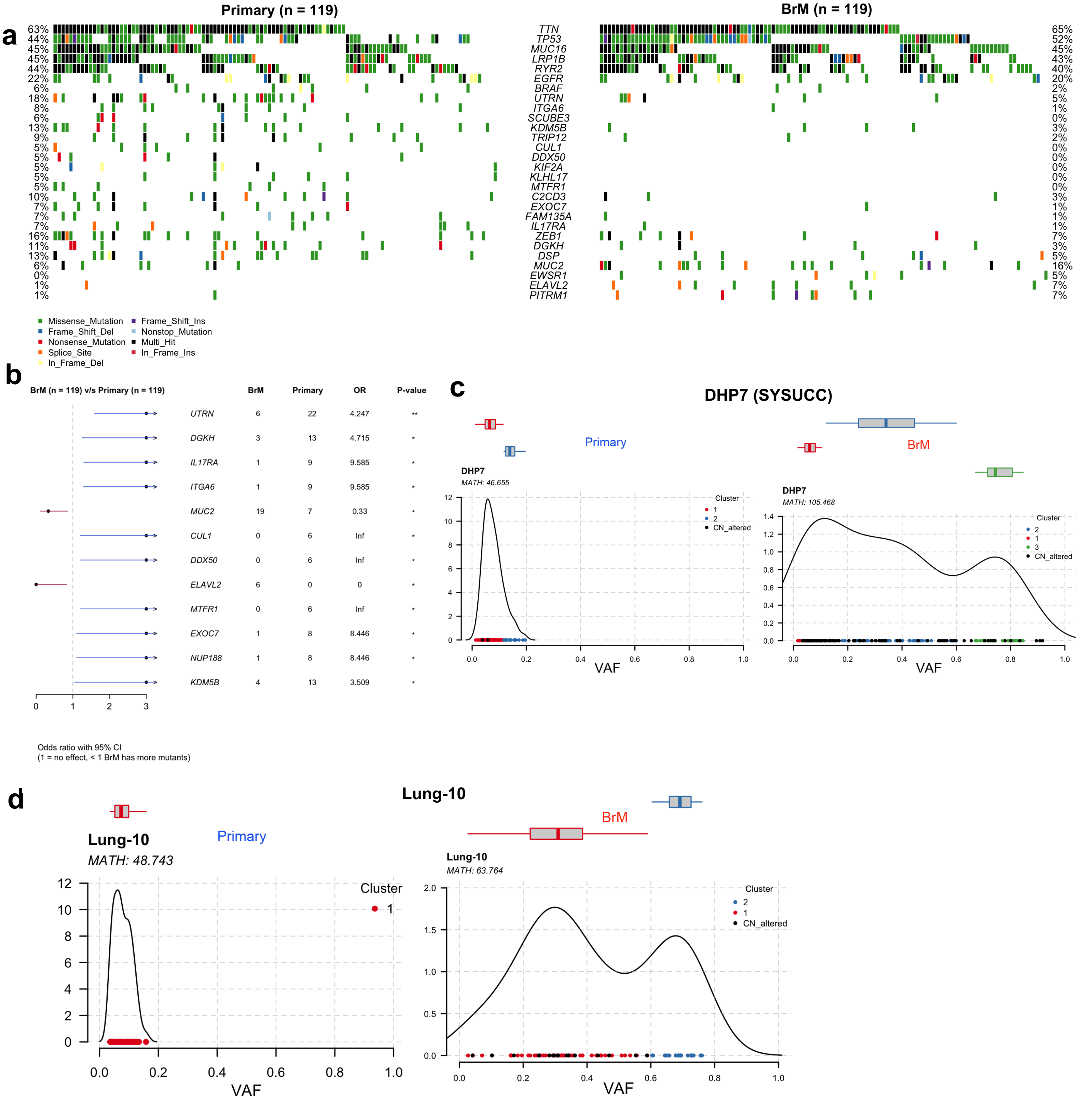


**Fig. S2. Related to Figure 2. Mutation landscape in paired primary lung cancer and brain metastases. a.** Oncoplot showing the specifically mutated genes in paired primary lung cancer and lung cancer brain metastasis (LC-BrM) specimens. **b.** Forrest plot to show the specifically mutated genes comparison in paired primary lung cancer and LC-BrM tumor specimens. **c and d.** Cluster plots of the primary lung cancer (left) and LC-BrM sample (right) derived from patient DHP7 and Lung-10. X-axis represented variant allele frequency, the top bar showed the number of clusters on top of each plot, and the MATH number was noted in the upper left corner.

# Supplemental Figure 3

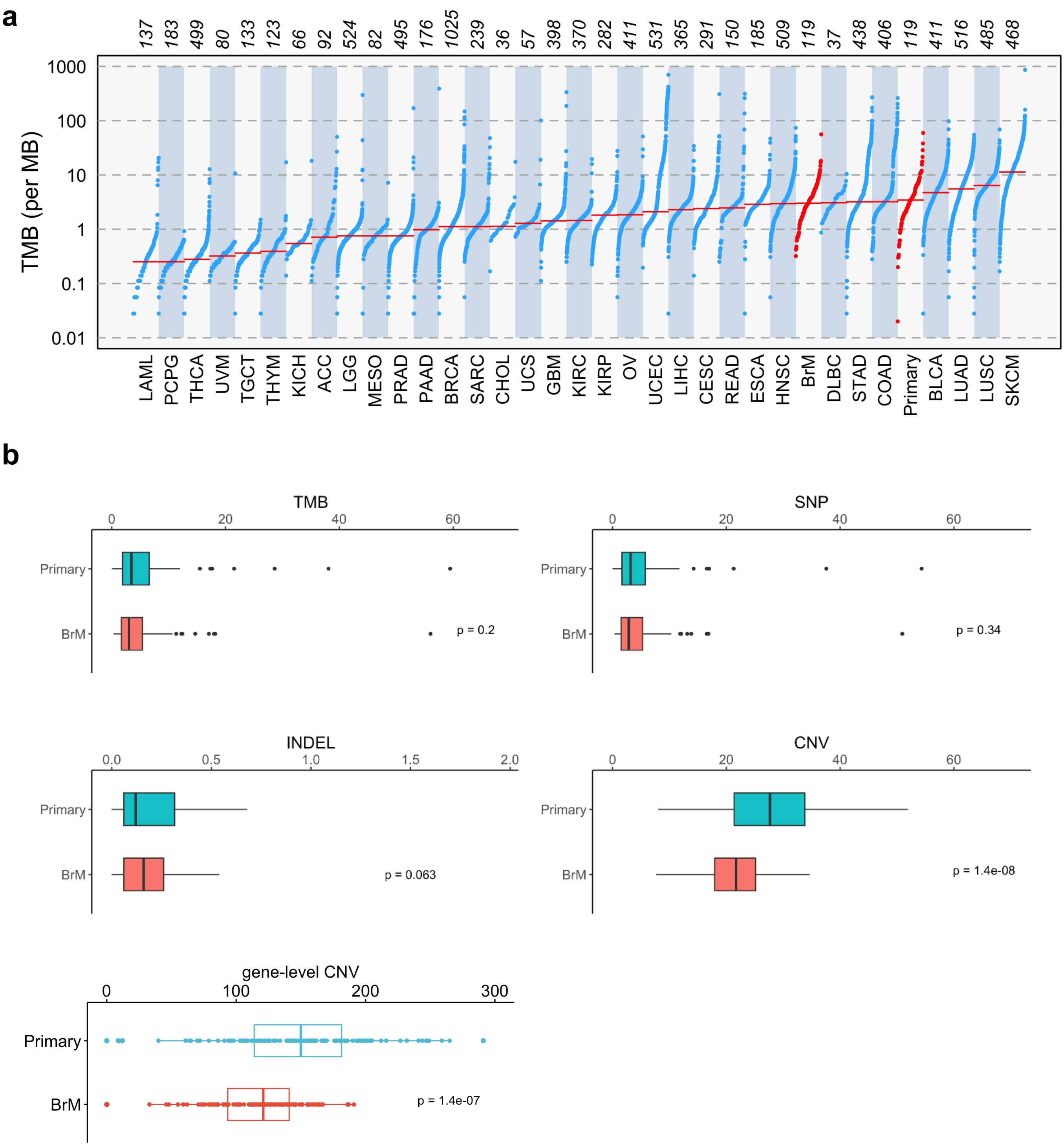


**Fig. S3. Related to Figure 3. Comparative tumor mutational burden in lung cancer and brain metastases. a**. Tumor mutation load comparison in primary, brain metastasis (BrM) against 33 TCGA cohorts derived from MC3 project. Top annotation is number of samples in each cohort, and y-axis is the logscale of tumor mutational burden with median mutation marked with red horizontal line. **b**. Box plots to show mutational burden comparison in matched primary lung cancer and BrM. The p-value was determined using pairwise two-sided Wilcoxon test.

# Supplemental Figure 4

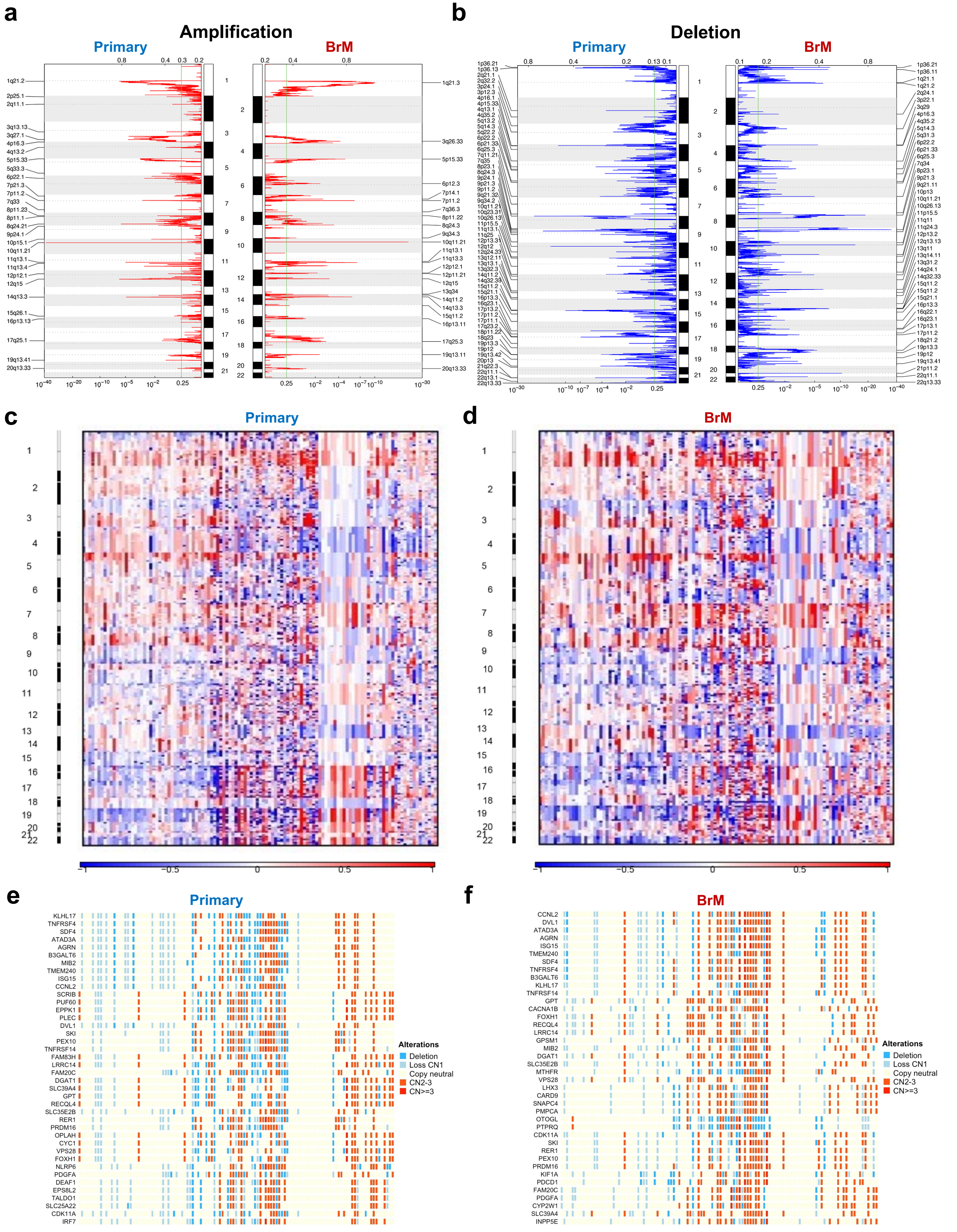

**Fig. S4. Related to Figure 4. Copy number alterations in primary lung cancer versus brain metastases**. **a and b.** Copy number gain (**a**) or loss (**b**) on the primary lung cancer (left column) and matched brain metastasis (BrM) samples (right column). **c and d**. Heatmap of copy number profiles for samples from primary lung cancer (**c**) and BrMs (**d**). Each row represents the copy number profile of a tumor sample across chromosomes 1 to 22 and X. Red indicates copy number gain; blue, loss. e and f. Specific genes of copy number gain and loss in primary lung cancer (**e**) and BrMs (**f**). Red indicates copy number gain (CN >= 3, CN2-3); yellow, no change (copy neutral); blue, loss (Deletion, Loss CN1).

# Supplemental Figure 5

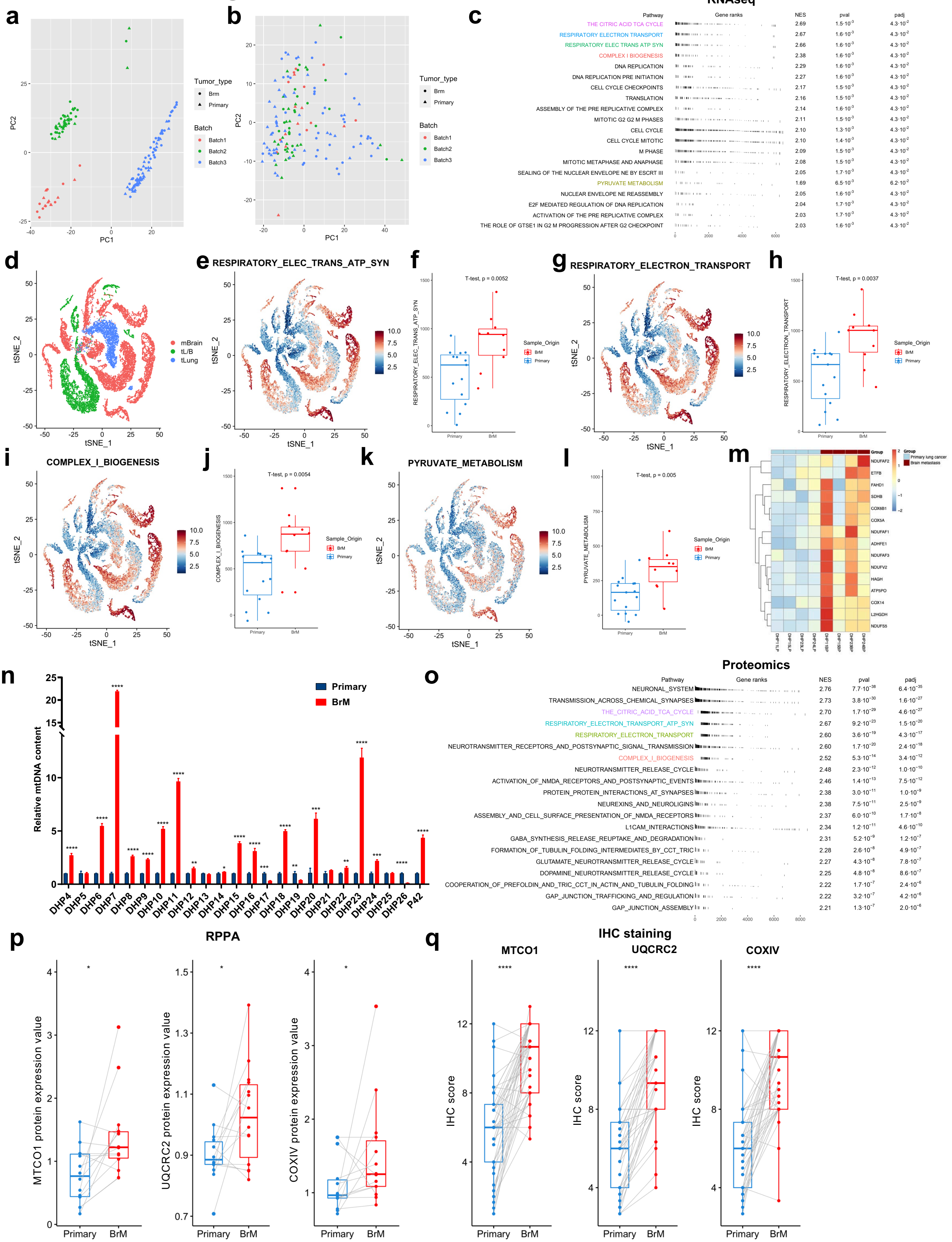

**Fig. S5. Related to Figure 5. Principal component analysis and pathway enrichment in lung cancer brain metastasis. a and b.** PCA plots to show the distribution of samples from two cohorts of 56 patients before (**a**) and after (**b**) ComBat process. **c.** The table of top 20 ranked gene pathways that were positively correlated with the lung cancer brain metastasis (LC-BrM) phenotype. Pathways were ordered by p-value and normalized enrichment score (NES). Five pathways related to mitochondrial biogenesis and oxidative phosphorylation were highlighted with different colors. **d.** tSNE plot of ~20,000 single epithelial cells with color-coded by three major cell lineages: 15,463 brain metastasis (mbrain) single cells, 7,270 early primary lung cancer (tLung) single cells and 6582 advanced-stage lung cancer (tL/B) single cells. **e-l**. tSNE plots and box plots of enrichment score of single epithelialcells for the rest four mitochondrial pathways mentioned in Figure 5b. **m.** the heatmap showing differentially expressed proteins related to mitochondrial biogenesis as identified in LC-BrMs in comparison with paired primary lung cancers. **n**. Relative mitochondrial DNA (mtDNA) content of paired primary lung cancer and brain metastasis lesions. The p-value was determined using pairwise two-sided Student's t test. **o.** The table of top 20 ranked protein pathways that were positively correlated with the LC-BrM phenotype. Pathways were ordered by p-value and NES. Four pathways related to mitochondrial biogenesis and oxidative phosphorylation were highlighted with different colors. **p.** Box plots showing the protein expression of MTCO1, UQCRC2 and COXIV based on reverse phase protein array data in nine paires of primary lung and brain metastasis lesions. The p-value was determined using pairwise two-sided Student's t test. **q.** Box plots for the immunohistochemistry scores of MTCO1, UQCRC2 and COXIV in 51 paires of primary lung and brain metastatic lesions. The p-value is determined using pairwise two-sided Student's t test. *, $p<0.05$; **, $p<0.01$; ***, $p<0.001$; ****, $p<0.0001$.

# Supplemental Figure 6

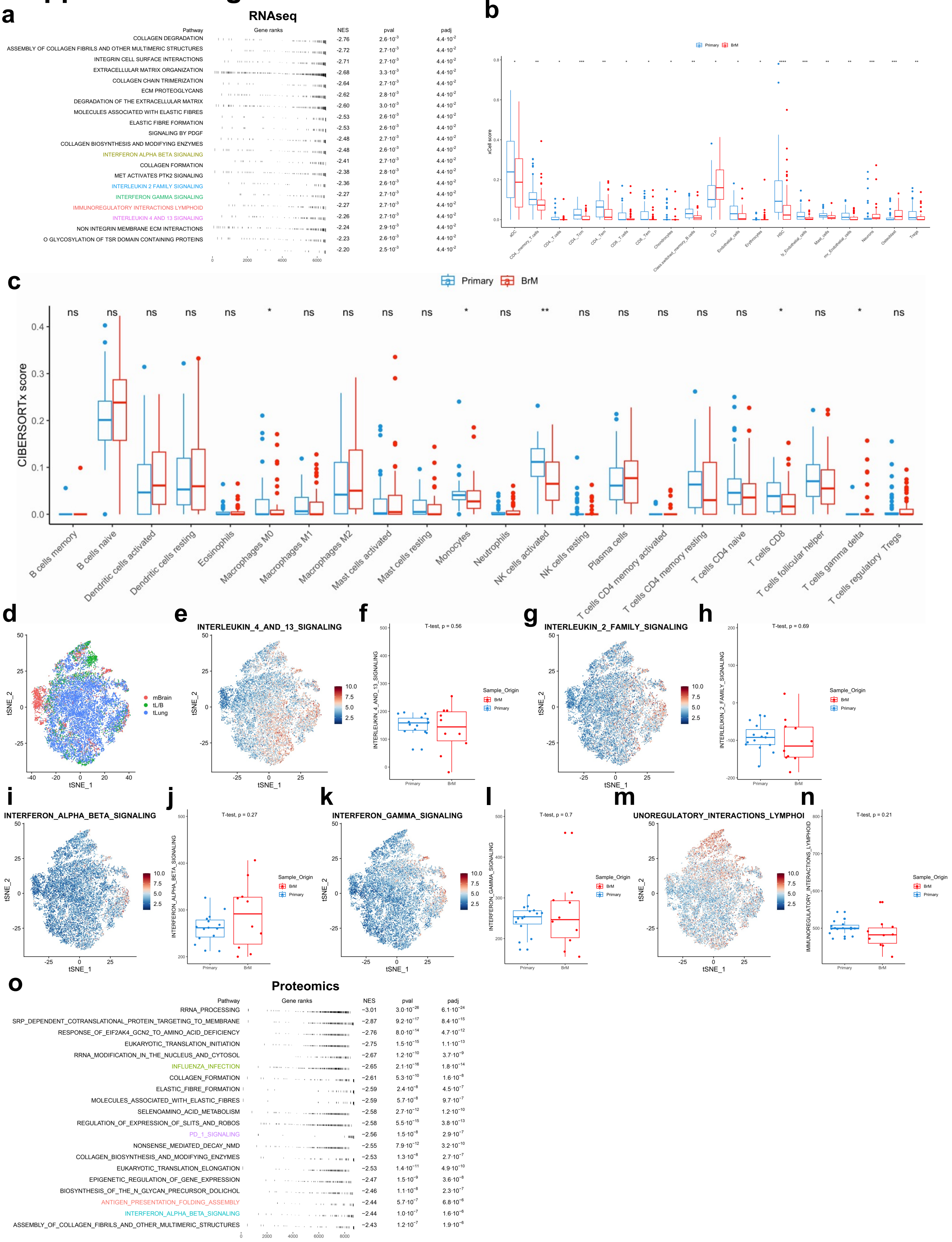

**Fig. S6. Related to Figure 6. Immune pathway correlations and lymphocyte composition in lung cancer brain metastases. a.** The table of bottom 20 ranked gene pathways that were negatively correlated with the phenotype of lung cancer brain metastasis (LC-BrM). Pathways were ordered by p-value and normalized enrichment score (NES). Five immune related pathways were highlighted with different colors. **b and c.** Box plot representation of xCell (**b**) and CIBERSORTx (**c**) scores for patients with paired primary lung cancer and LC-BrMs. The p-value was determined by use of pairwise t-test. **d.** tSNE plot of ~20,000 lymphocytes with color-coded by three major cell lineages: 3910 brain metastasis (mbrain) single cells, 18587 early primary lung cancer (tLung) single cells, and 3627 advanced-stage lung cancer (tL/B) single cells. **e-n.** tSNE plots and box plots of enrichment score of single lymphocytes for the five immune pathways mentioned in Figure 6a. **o**. The table of bottom 20 ranked protein pathways that were negatively correlated with the phenotype of LC-BrM. Pathways were ordered by p-value and NES. Four immune related pathways were highlighted with different colors.

# Supplemental Figure 7

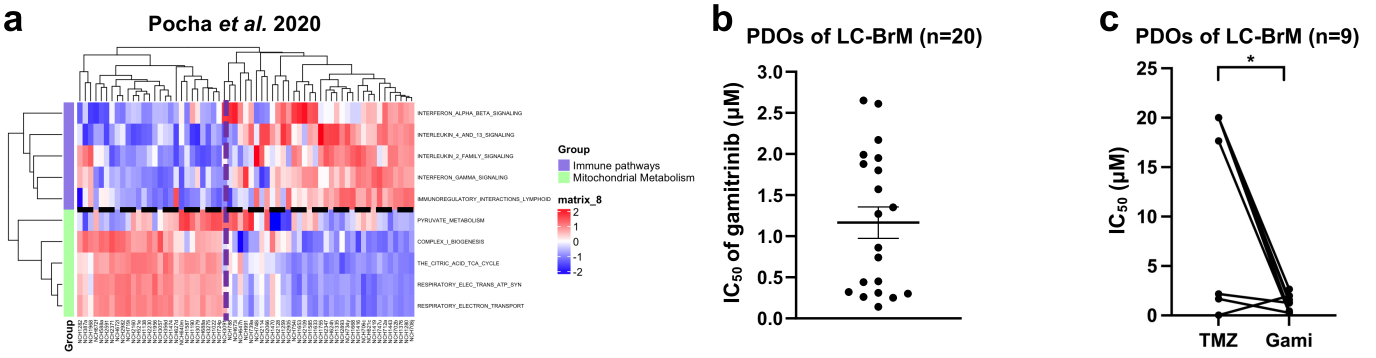


**Fig. S7. Related to Figure 7. Enrichment analysis of mitochondrial and immune pathways in independent brain metastasis cohort. a.** The heatmap showing the enrichment of indicated five mitochondrial pathways and five immune pathways in an independent cohort of 63 brain metastasis (BrM) lesions with mRNA microarray data. **b.** Scatter plot showing the $IC_{50}$ to gamitrinib of 20 BrM patient-derived organoids (PDOs). **c.** Box plots showing the $IC_{50}$ of temozolomide (TMZ) and gamitrinib in BrM PDOs. The p-value was determined using pairwise two-sided Wilcoxon test. *, $p<0.05$.

# Supplemental Figure 8

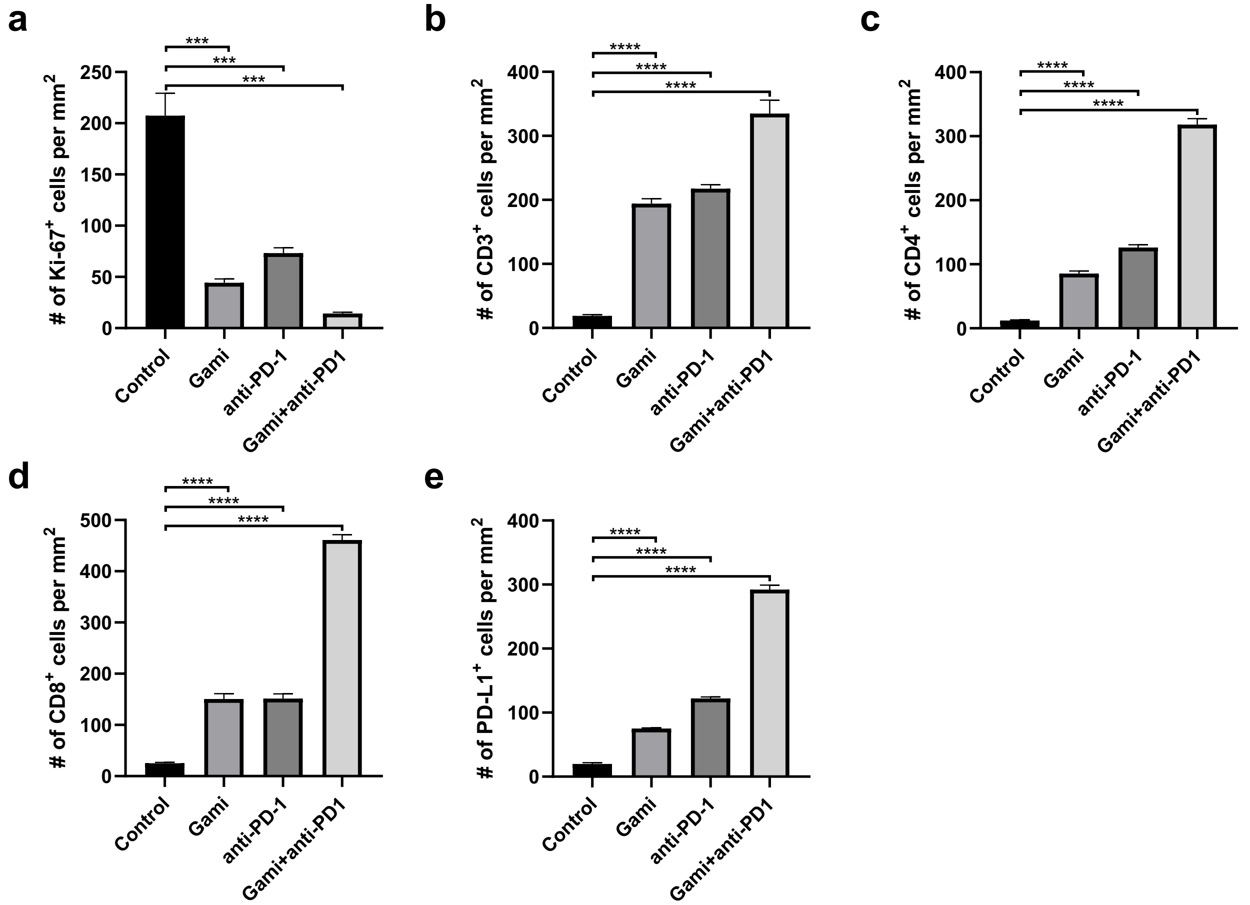


**Fig. S8. Related to Figure 8. Immunohistochemical profiling of proliferation and immune markers in brain metastases. a-e.** Bar plots of quantification analyses of immunohistochemistry staining of Ki-67 (**a**), CD3 (**b**), CD4 (**c**), CD8 (**d**), and PD-L1 (**e**) in Lewis lung carcinoma brain metastases. The p-value is determined using two-sided Student's t test. ***, p<0.001; ****, p<0.0001.
